# MicroRNA-216a Promotes Endothelial Inflammation by Smad7/I*κ*B*α* Pathway in Atherosclerosis

**DOI:** 10.1155/2020/8864322

**Published:** 2020-11-17

**Authors:** Shujun Yang, Yu Chen, Xuenan Mi, Shuyuan Zhang, Yunyun Yang, Rutai Hui, Weili Zhang

**Affiliations:** ^1^State Key Laboratory of Cardiovascular Disease, FuWai Hospital, National Center for Cardiovascular Diseases, Peking Union Medical College & Chinese Academy of Medical Sciences, Beijing, China; ^2^Xiamen Cardiovascular Hospital, Xiamen University, Xiamen, China

## Abstract

**Background:**

The endothelium is the first line of defence against harmful microenvironment risks, and microRNAs (miRNAs) involved in vascular inflammation may be promising therapeutic targets to modulate atherosclerosis progression. In this study, we aimed to investigate the mechanism by which microRNA-216a (miR-216a) modulated inflammation activation of endothelial cells*. Methods*. A replicative senescence model of human umbilical vein endothelial cells (HUVECs) was established, and population-doubling levels (PDLs) were defined during passages. PDL8 HUVECs were transfected with miR-216a mimics/inhibitor or small interfering RNA (siRNA) of SMAD family member 7 (Smad7). Real-time PCR and Western blot assays were performed to detect the regulatory role of miR-216a on Smad7 and NF-*κ*B inhibitor alpha (I*κ*B*α*) expression. The effect of miR-216a on adhesive capability of HUVECs to THP-1 cells was examined. MiR-216a and Smad7 expression *in vivo* were measured using human carotid atherosclerotic plaques of the patients who underwent carotid endarterectomy (*n* = 41).

**Results:**

Luciferase assays showed that Smad7 was a direct target of miR-216a. Smad7 mRNA expression, negatively correlated with miR-216a during endothelial aging, was downregulated in senescent PDL44 cells, compared with young PDL8 HUVECs. MiR-216a markedly increased endothelial inflammation and adhesive capability to monocytes in PDL8 cells by promoting the phosphorylation and degradation of I*κ*B*α* and then activating NF-*κ*B signalling pathway. The effect of miR-216a on endothelial cells was consistent with that blocked Smad7 by siRNAs. When inhibiting endogenous miR-216a, the Smad7/I*κ*B*α* expression was rescued, which led to decreased endothelial inflammation and monocytes recruitment. In human carotid atherosclerotic plaques, Smad7 level was remarkably decreased in high miR-216a group compared with low miR-216a group. Moreover, miR-216a was negatively correlated with Smad7 and I*κ*B*α* levels and positively correlated with interleukin 1 beta (IL1*β*) expression *in vivo*.

**Conclusion:**

In summary, our findings suggest a new mechanism of vascular endothelial inflammation involving Smad7/I*κ*B*α* signalling pathway in atherosclerosis.

## 1. Introduction

Atherosclerosis, a chronical inflammatory disorder, is a critical pathogenesis underlying cardiovascular diseases. Endothelial inflammation and dysfunction play a pivotal role in various stages of atherosclerosis, which can be induced by conventional risk factors such as aging, obesity, smoking, hypertension, hyperglycaemia, and hyperlipidemia [1]. Except for these risk factors, a growing body of evidence indicates that microRNAs (miRNAs) are potentially epigenetic factors in regulating the progression of vascular endothelial senescence, inflammatory response, and atherosclerosis [2, 3].

The miRNAs, single-stranded, small noncoding RNAs comprising of approximately 22 nucleotides can bind to the complementary sequence in the 3′ untranslated region (3′UTR) of their specific target messenger RNAs (mRNAs) and regulate the translational suppression or destabilization of these mRNAs [4]. MicroRNA-216a (miR-216a) is highly conserved among various species including human, mouse, rat, and zebrafish. Several studies have demonstrated that miR-216a has important roles in vascular endothelial aging, inflammation, and autophagy defects in atherosclerosis [5–7]. Our previous work showed that plasma miR-216a level increases in aged patients with coronary artery disease compared with healthy controls; experimental results further showed that stable expression of miR-216a can contribute to a premature senescence-like phenotype in human endothelial cells, inhibit endothelial proliferation, and activate inflammatory responses by inhibiting SMAD family member 3 (Smad3) expression [5]. The miR-216a overexpression is also involved in the loss of autophagic function by targeting beclin 1 during endothelial cell aging [6]. Wang et al. further reported that miR-216a-mediated repressive effects on autophagy and survival can be abrogated by the long noncoding RNA (lncRNA) MALAT1 in human endothelial cells [7].

Besides, miR-216a plays important regulatory roles in macrophage polarization and foam cells formation during atherosclerosis development [8, 9]. Our previous study found that miR-216a can promote M1 macrophage polarization and lipid uptake ability *via* targeting Smad3/NF-*κ*B pathway *in vitro* and accelerate atherosclerotic plaque development in a mouse model [8]. Plasma miR-216a is highly expressed in coronary artery disease patients with venerable plaques compared with the patients with stable plaques, indicating that miR-216a may be a potential biomarker for atherosclerotic diseases [8]. Gong et al. reported that miR-216a motivates cholesterol efflux by targeting cystathionine gamma-lyase (CSE) in macrophage cells [9].

The effects of miR-216a on endothelial functions may be differential due to the alternative targeting genes under certain microenvironments. The bioinformatics prediction analysis showed that there is a potential miR-216a-binding site within the 3′UTR of SMAD family member 7 (Smad7) gene. Smad7, a major negative inhibitor of transforming growth factor beta (TGF*β*) signal pathway, plays complex regulatory roles in inflammatory processes. Some studies showed that Smad7 can block the protective, anti-inflammatory effect of TGF*β via* regulating the Smad2/3 expression and subsequently inducing NF-*κ*B activation in human intestinal lamina propria mononuclear cells or in macrophages [10, 11]. However, inconsistent findings existed. For example, Smad7 retains the NF-*κ*B inhibitor alpha (I*κ*B*α*) expression; inhibits NF-*κ*B-activated genes such as tumour necrosis factor alpha (TNF*α*), interleukin 1 beta (IL1*β*), and intercellular adhesion molecule 1 (ICAM1); and thereafter plays an anti-inflammatory role in several tumour cell lines [12, 13].

To date, the molecular mechanisms of miR-216a in vascular endothelial cells have not been comprehensively explored. In this study, we aimed to investigate whether miR-216a could modulate the activation and inflammatory responses of endothelial cells by targeting Smad7 *in vitro* and further examined the relationship between miR-216a and Smad7 expression *in vivo* in human carotid atherosclerotic plaques.

## 2. Materials and Methods

### 2.1. Cell Culture and miRNA or siRNA Transfection

Primary human umbilical vein endothelial cells (HUVECs) were cultured in endothelial cell medium (ScienCell, San Diego, CA, USA) containing 5% fetal bovine serum and 1% penicillin/streptomycin, and the replicative senescence model was established. Population-doubling levels (PDLs) were calculated during the passages according to an equation reported previously (1), in which Ch is the number of cells at harvest and Cs is the number of cells seeded [14]. Consequently, PDL8 and PDL44 HUVECs were defined as young and aging cells, respectively.

(1) $$\begin{array}{c} \text{PD}=\log_{2}\left(\frac{\text{Ch}}{\text{Cs}}\right). \end{array}$$

To explore the regulatory role of miR-216a in target gene expression, PDL8 HUVECs were transfected with 100 nM miR-216a mimics or miR-216a inhibitor (GenePharma, Suzhou, China) using the lipofectamine 3000 reagent (Invitrogen, Carlsbad, CA, USA) and harvested at 48 h posttransfection. To determine the effects of Smad7 on endothelial cells, Smad7 was silenced *via* transfection of 40 nM small interfere RNAs (siRNAs) (Invitrogen, Carlsbad, CA, USA). The following siRNA of Smad7 was used: sense 5′ CAGCGGCCCAAUGACCACGAGUUUA 3′, and antisense 5′ UAAACUCGUGGUCAUUGGGCCGCUG 3′.

### 2.2. Assays for Endothelial Cell Adhesion Activity

The capacity of endothelial adhesion to monocytes was examined in PDL8 HUVECs. Confluent endothelial monolayers were established in 24-well plate before incubation for 48 h with miR-216a mimics/inhibitor or Smad7 siRNA in the presence of lipofectamine 3000 reagent. THP-1 monocytes (Zhong Qiao Xin Zhou Biotechnology, Shanghai, China) were labelled with CellTracker™ CM-Dil (Life Technologies, Carlsbad, CA, USA) and added to 24-well plates containing HUVEC monolayers (1 × 10^5^ THP-1 cells/well). After incubation at 37°C for 30 min, cells were gently washed twice with PBS to remove nonadherent monocytes. The number of THP-1 cells attached to HUVEC surface was quantified throughout 5 random fields.

### 2.3. Real-Time PCR for mRNA and miRNA Expression Analysis

The mRNA expression of ICAM1, IL1*β*, and Smad7 was analysed by real-time PCR. The total RNAs of HUVECs were extracted by TRIzol reagent (Invitrogen, Carlsbad, CA, USA) in accordance with the manufacturer's instruction; then, 1 *μ*g RNA was used to synthesize cDNA using PrimeScript Reverse Transcriptase assay (Takara, Dalian, China). Real-time PCR was performed with SYBR Green qPCR mix (YEASEN, Shanghai, China) utilizing the ABI ViiA7™ System with 384-Well Block (Applied Biosystems, Foster City, CA, USA). A relative expression was determined by the *ΔΔ*Ct method, and GAPDH gene was applied as the internal reference. Primers for real-time PCR were listed as following: ICAM1 forward 5′ TCTGTGTCCCCCCTCAAAAGTC 3′and reverse 5′ GGGTCTCTATGCCCAACAA 3′; IL1*β* forward 5′ CAGCTACGAATCTCCGACCAC 3′ and reverse 5′ GGCAGGGAACCAGCATCTTC 3′; Smad7 forward 5′ ACTCCAGATACCCGATGGATTT 3′ and reverse 5′ CCTCCCAGTATGCCACCAC 3′; GAPDH forward 5′ GAAGGTGAAGGTCGGAGTCA 3′ and reverse 5′ GGAAGATGGTGATGGGATTTC 3′.

Total RNAs extracted from HUVECs or human carotid plaque tissues by Trizol reagent were used to examine the miR-216a expression using miScript II reverse transcription kit and miScript SYBR Green PCR kit (QIAGEN, Hilden, Germany) by real-time PCR on the ABI ViiA7™ System. The miR-216a level was normalized to U6 small nuclear RNA.

### 2.4. Luciferase Reporter Assay

There exists a putative miR-216a binding sequence between 1,230 and 1,236 base pairs (bps) in the 3′UTR of Smad7 (ENSG00000101665). Primers were designed to amplify the Smad7 3′UTR sequence: forward 5′ GCCAAGCTTTCTTCTTCTCGTCCTCGTT 3′ and reverse 5′ GCCGAGCTCTAGGTGATAACACCCATAGA 3′. The Smad7 3′UTR amplicons, with the length of 1,134 bps, were inserted into pMIR-REPORT™ Luciferase vectors (Ambion, Austin, TX, USA).

To determine the regulatory effect of miR-216a on the mRNA expression of Smad7, the luciferase reporter assays were performed in HEK293T cells (China Infrastructure of Cell Line Resources, Beijing, China). Cells were cotransfected with 100 ng Smad7 3′UTR or 3′UTR mutant plasmid in 96-well plates and 50 nM miR-216a mimics or negative control (NC) (*n* = 8 per group) using Lipofectamine 3000 (Invitrogen, Carlsbad, CA, USA). After 2 days, the firefly and renilla luciferase activities were detected with the Dual Luciferase Reporter Assay System (Promega, Madison, WI, USA) utilizing an UniCel DxC 800 Synchron Analyzer (Backman, CA, USA). Renilla luciferase activity was used to normalize firefly luciferase activity.

### 2.5. Western Blot Analysis

The role of miR-216a in protein expression of Smad7, I*κ*B*α*, and phosphorylated I*κ*B*α* was investigated using Western blot. PDL8 HUVECs were lysed by RIPA lysis buffer (Beyotime Biotechnology, Shanghai, China) containing both of protease and phosphatase inhibitors (Roche, Mannheim, Germany). Protein concentrations of cell lysates were assessed by the Pierce BCA Protein Assay Kit (Invitrogen, Carlsbad, CA, USA). For each sample, 50 *μ*g of protein was loaded and electrophoresed using 10% SDS-PAGE gel and subsequently transferred to the nitrocellulose membrane (Millipore, MA, USA). After blocked by 5% BSA, membranes were incubated with primary antibodies and then corresponding secondary antibodies. Primary antibodies were rabbit polyclonal anti-Smad7 (Proteintech, Chicago, IL, USA), anti-I*κ*B*α*, anti-Phospho-I*κ*B*α* (Ser32), and anti-GAPDH (Cell Signaling Technology, USA), in which GAPDH was used as an internal reference. Secondary antibody was anti-rabbit (1 : 5000) labelled with horseradish peroxidase. The protein bands were visualized using the FluorChem™ R System (ProteinSimple, CA, USA), and proteins were quantified by the AlphaView Software.

### 2.6. Human Carotid Atherosclerotic Plaques

The human carotid atherosclerotic plaques were obtained from patients undergoing the carotid endarterectomy surgery (*n* = 41), and clinical characteristics of patients are shown in Table 1. Tissues were minced and embedded into RNAlater™ Stabilization Solution (Ambion, Carlsbad, CA, USA), then stored at -80°C.This study was approved by the ethics committee of the FuWai Hospital.

### 2.7. Statistical Analysis

The quantitative variables were presented as means ± S.D. or medians and interquartile ranges (IQRs), while the categorical variables were quantified as absolute counts (percentages). Group differences were analysed by Student *t*-test, one-way ANOVA, or Mann–Whitney nonparametric test for the quantitative variables, where appropriate. The *χ*^2^ test was utilized to compare the categorical variables. The Pearson's correlation analysis was performed to evaluate the correlation between miR-216a levels and the mRNA expression of Smad7, I*κ*B*α*, and IL1*β* in human atherosclerotic plaques. The SPSS Statistics 20.0 software (SPSS Inc, Chicago, USA) was used, and statistical significance was considered when *P* < 0.05.

## 3. Results

### 3.1. Smad7 Was a Direct Target of miR-216a

**Figure 1:**
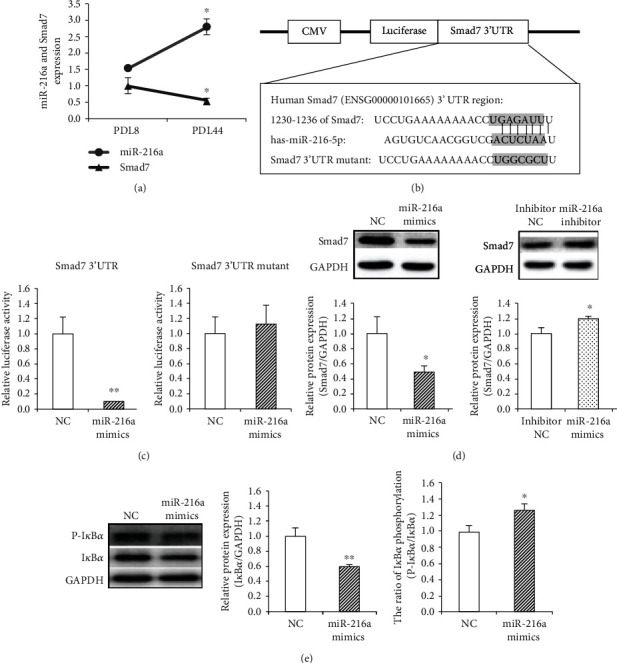
Smad7 was a direct target of miR-216a. (a) Compared with young PDL8 HUVECs, senescent PDL44 cells exhibited upregulated miR-216a and downregulated Smad7 (*n* = 5). (b) Schematic diagram of the potential binding site of miR-216a within Smad7 3′UTR and the luciferase plasmids. (c) miR-216a inhibited the luciferase activity of Smad7 3′UTR vector but not Smad7 3′UTR mutant vector in HEK293T cells (*n* = 8). (d) Overexpression of miR-216a suppressed the Smad7 protein expression; conversely, miR-216a inhibitor upregulated Smad7 protein level in PDL8 cells (*n* = 5). (e) The total protein expression of I*κ*B*α* was reduced, and the proportion of I*κ*B*α* phosphorylation (phosphorylated I*κ*B*α*/total I*κ*B*α*) was increased in PDL8 cells with transfection of miR-216a (*n* = 5). ^∗^*P* < 0.05, ^∗∗^*P* < 0.01.

Compared with that expression in young PDL8 HUVECs, Smad7 mRNA was found to be downregulated by 47% (*P* = 0.03) in senescent PDL44 cells, which was negatively correlated with the miR-216a expression during endothelial aging (Figure 1(a)). The computational miRNA target analysis by TargetScan Release 7.1 from miRNA databases indicated that Smad7 was a potential target gene of miR-216a, containing a candidate miR-216a binding site between 1,230 and 1,236 bps in the 3′UTR (Figure 1(b)). Next, the luciferase reporter vectors of Smad7 3′UTR and 3′UTR mutant were constructed. Cotransfection of miR-216a mimics with Smad7 3′UTR vector inhibited the luciferase activity by 91% in HEK293T cells (*P* < 0.001), whereas the luciferase reporter carrying Smad7 3′UTR mutant did not respond to regulation of miR-216a (Figure 1(c)).

The regulatory role of miR-216a in the Smad7 protein expression was assessed in PDL8 HUVECs. Overexpression of miR-216a resulted in remarkable downregulation of the Smad7 protein expression by 50% (*P* = 0.02), while antagomiR-216a, a synthetic inhibitor of endogenous miR-216a, significantly increased the Smad7 protein expression by 19% (*P* = 0.03; Figure 1(d)). Furthermore, miR-216a overexpression enhanced I*κ*B*α* phosphorylation by 27% (*P* = 0.01) and subsequently reduced the I*κ*B*α* protein level by 41% (*P* = 0.004; Figure 1(e)), which indicated the proinflammatory role of miR-216a in downregulating Smad7 and I*κ*B*α* in endothelial cells.

### 3.2. miR-216a Promoted Monocytes Adhesion to Endothelial Cell Monolayers

**Figure 2:**
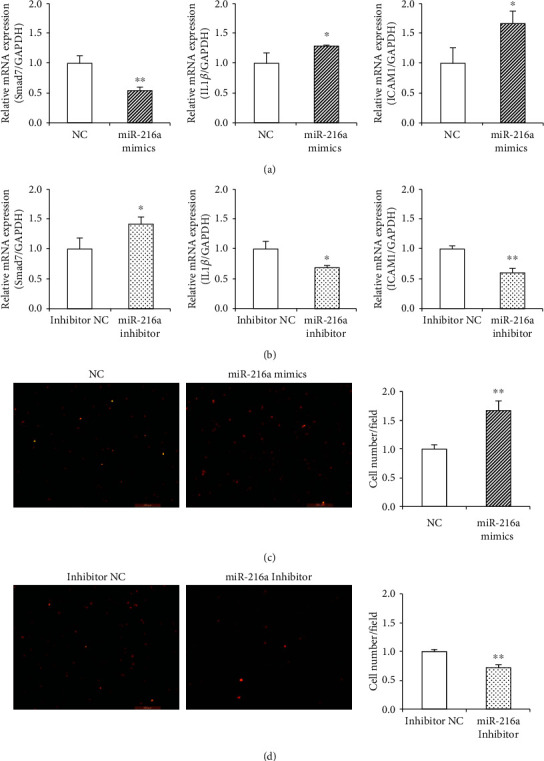
miR-216a promoted monocytes adhesion to HUVEC monolayers. (a, b) Smad7 mRNA expression was inhibited by miR-216a mimics and increased by miR-216a inhibitor. Simultaneously, mRNA levels of IL1*β* and ICAM1 were upregulated by miR-216a and inhibited by miR-216a inhibitor in PDL8 HUVECs. (c, d) The endothelial adhesion ability of PDL8 HUVECs to THP-1 monocytes was increased by miR-216a mimics, whereas decreased by miR-216a inhibitor. Scale bars: 200 *μ*m. *n* = 5 for each group; ^∗^*P* < 0.05, ^∗∗^*P* < 0.01.

To investigate whether miR-216a regulated endothelial inflammation by targeting Smad7, the mRNA expression levels of IL1*β* and ICAM1 were examined in PDL8 HUVECs. The results showed that miR-216a overexpression increased the IL1*β* and ICAM1 expression by 29% (*P* = 0.04) and 66% (*P* = 0.03) (Figure 2(a)), while miR-216a inhibition suppressed the mRNA expression levels of IL1*β* and ICAM1 by 33% (*P* = 0.002) and 41% (*P* = 0.05), respectively (Figure 2(b)). The THP-1 cell adhesion assay showed that miR-216a increased the adhesion ability of THP-1 monocytes to endothelial cells by 66% (*P* = 0.004) in PDL8 HUVECs with transfection of miR-216a mimics (Figure 2(c)), whereas inhibition of endogenous miR-216a reduced endothelial adhesion ability to THP-1 cells by 27% (*P* = 0.003; Figure 2(d)).

### 3.3. miR-216a Enhanced Endothelial Inflammation *via* Smad7/I*κ*B*α* Pathway

**Figure 3:**
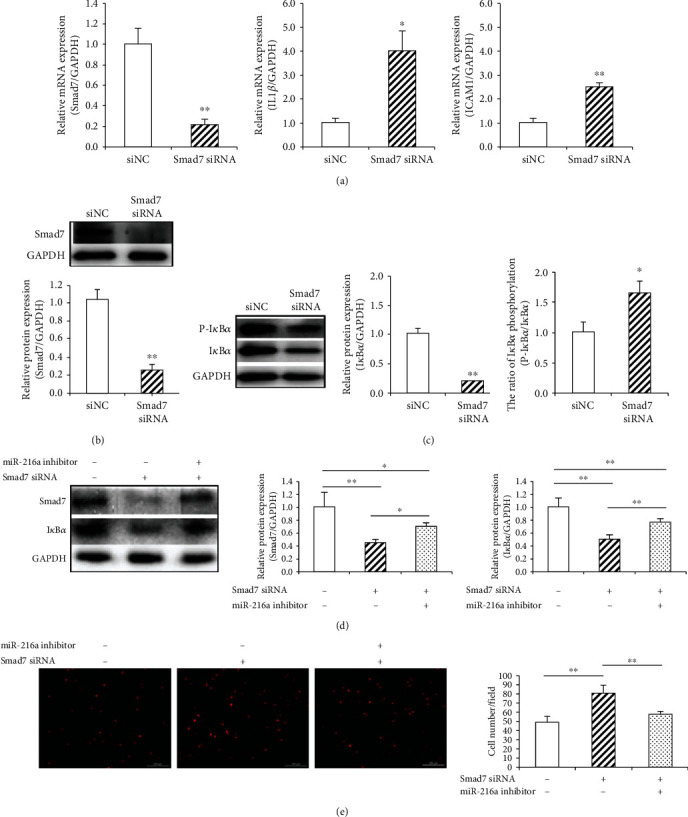
miR-216a enhanced endothelial inflammation *via* Smad7/I*κ*B*α* pathway. (a) Knockdown of Smad7 with siRNA resulted in the downregulation of the Smad7 mRNA expression and the remarkable upregulation of IL1*β* and ICAM1 mRNA levels in PDL8 HUVECs. (b) Smad7 protein level in HUVECs was inhibited by its siRNA. (c) The total I*κ*B*α* expression was reduced, and the proportion of I*κ*B*α* phosphorylation was increased after silencing Smad7. (d) The protein expression of Smad7 and I*κ*B*α* was rescued by miR-216a inhibitor when endogenous Smad7 was silenced. (e) The capacity of endothelial adhesive to THP-1 monocytes was increased by miR-216a inhibitor after silencing Smad7. Scale bars: 200 *μ*m. *n* = 5 for each group; ^∗^*P* < 0.05, ^∗∗^*P* < 0.01.

As mentioned above, miR-216a was found to downregulate the Smad7 expression, while simultaneously increasing the expression of NF-*κ*B response genes like IL1*β* and ICAM1, which subsequently enhances the endothelial adhesive ability to monocytes. To further investigate the molecular mechanism by which miR-216a regulated endothelial inflammation *via* the target Smad7, siRNAs of Smad7 were transfected into PDL8 cells. It was found that the Smad7 mRNA expression was significantly suppressed by 78% (*P* = 0.01) after inhibition of endogenous Smad7 by siRNAs, while the IL1*β* and ICAM1expression increased remarkably by 4.0-fold (*P* = 0.03) and 2.5-fold (*P* = 0.007), respectively (Figure 3(a)). The Smad7 protein expression was downregulated considerably by 75% (*P* = 0.002; Figure 3(b)); subsequently, the phosphorylation level of I*κ*B*α* was increased by 66% (*P* = 0.01), and the total protein level of I*κ*B*α* was reduced by 79% (*P* < 0.001) in HUVECs when silencing Smad7 (Figure 3(c)). These results supported that Smad7 plays an anti-inflammatory role through inhibiting the phosphorylation and degradation of I*κ*B*α* in endothelial cells.

Next, endogenous miR-216a and Smad7 in HUVECs were inhibited by cotransfection with miR-216a inhibitor and Smad7 siRNA. We found that miR-216a inhibition rescued the downregulation of Smad7 and I*κ*B*α* protein levels by 58% (*P* = 0.05) and 56% (*P* = 0.003), respectively (Figure 3(d)). Under the condition of silencing Smad7, the endothelial adhesive ability to monocytes was suppressed by miR-216a inhibitor by 30% (*P* < 0.001; Figure 3(e)). All these results suggested that miR-216a promoted endothelial inflammation by directly regulating the Smad7/I*κ*B*α* pathway.

### 3.4. miR-216a Was Negatively Related with Smad7 Level in Human Atherosclerotic Plaques

**Figure 4:**
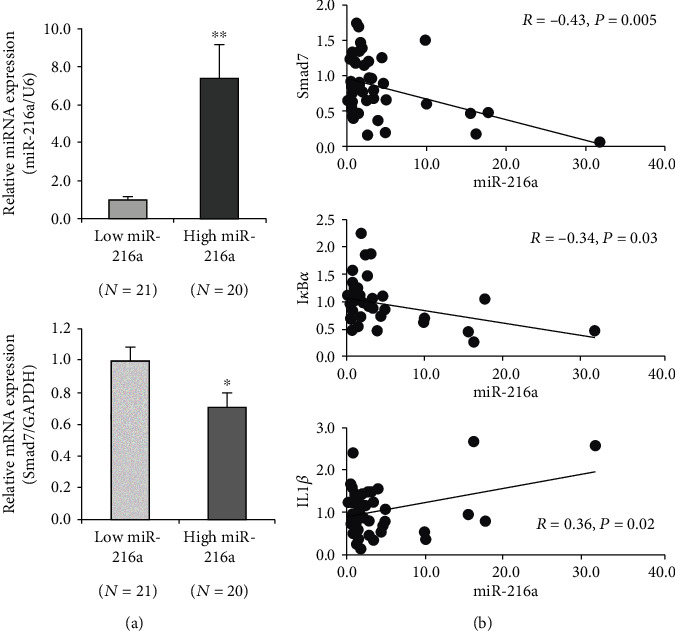
miR-216a was negatively related with the Smad7 expression in human atherosclerotic plaques. (a) The patients undergoing the carotid endarterectomy were stratified into two groups according to the median of the miR-216a expression in their carotid plaques (*n* = 41). The Smad7 expression was downregulated in the high miR-216a group (*n* = 20), compared to the low miR-216a group (*n* = 21). (b) miR-216a was negatively correlated with Smad7 and I*κ*B*α*, whereas positively correlated with IL1*β* in human carotid atherosclerotic plaques by Pearson's correlation analysis. ^∗^*P* < 0.05, ^∗∗^*P* < 0.01.

The regulatory effect of miR-216a on endothelial inflammation suggested a significant role of miR-216a in the pathogenesis of atherosclerosis. To assess the impact of miR-216a *in vivo*, miR-216a and Smad7 mRNA levels were examined in atherosclerotic plaques from the patients who underwent the carotid endarterectomy. The patients were stratified into the low miR-216a group (*n* = 21) or high miR-216a group (*n* = 20), according to the median of miR-216a expression level (1.91, IQR, 0.80-4.14). Compared with that expression in the low miR-216a group, miR-216a levels increased by 7.4-fold (*P* < 0.001), and the Smad7 mRNA expression markedly decreased by 30% (*P* = 0.02) in the high miR-216a group (Figure 4(a)). Moreover, we found that miR-216a was negatively correlated with Smad7 (*R* = −0.43, *P* = 0.005) and its downstream I*κ*B*α* (*R* = −0.34, *P* = 0.03) based on Pearson's correlation analysis. On the contrary, miR-216a was positively correlative with IL1*β* (*R* = 0.36, *P* = 0.02) (Figure 4(b)). These results were indicative of the regulatory effect of miR-216a on Smad7/I*κ*B*α* pathway in human atherosclerotic plaques.

## 4. Discussion

In the current study, we provided *in vitro* evidence that miR-216a promoted endothelial inflammation and the adhesion ability of monocytes to endothelial cells by directly targeting Smad7/I*κ*B*α* signalling pathway and subsequently increasing expression of NF-*κ*B response genes such as IL1*β* and ICAM1. In human carotid atherosclerotic plaque tissues, the Smad7 mRNA expression markedly decreased in the high miR-216a group in comparison with that in the low miR-216a group. Our findings extended the knowledge of miR-216a on endothelial inflammation involving Smad7/I*κ*B*α* pathway *in vitro* and *in vivo*.

The miR-216a may exert various effects on endothelial functions according to its different target genes. Our previous work showed that miR-216a induces endothelial aging and promotes inflammatory process *via* directly inhibiting Smad3/I*κ*B*α* signalling pathway [5]. In this study, bioinformatics analysis predicted that Smad7 might be an alternative target gene of miR-216a. Our further experiments confirmed Smad7 as a direct target gene of miR-216a; overexpression of miR-216a remarkably downregulated the mRNA and protein expression of Smad7, while inhibition of miR-216a significantly upregulated the expression of Smad7 in endothelial cells.

Smad7 is not only the major negative inhibitor of TGF*β* signal pathway but also functions as a key mediator of the crosstalk between proinflammatory and anti-inflammatory pathways. Smad2/3 signalling pathway is known as the canonical mediator of TGF*β* and responsible for its anti-inflammatory effect. Smad7 can form a stable complex with TGF*β* type I receptor and exert a proinflammatory effect; the underlying mechanism of which is to inhibit Smad2/3 pathway, reduce I*κ*B*α* expression, and then result in NF-*κ*B activation in human intestinal lamina propria mononuclear cells or in macrophages [10, 11]. On the other hand, Smad7 also exerts an anti-inflammatory effect by inhibiting phosphorylation of I*κ*B*α* and thus suppressing NF-*κ*B-activated genes in human head and neck squamous cell carcinoma lines or in mouse skin [12, 13]. To date, the effects of Smad7 have been extensively studied in inflammatory bowel diseases and tumour cells, but its role in vascular cells and atherosclerosis is still unclear. A recent study reported that low expression of Smad7 leads to activation of NF-*κ*B pathway and upregulation of proinflammatory cytokines (such as TNF*α* and IL1*β*) in human vascular smooth muscle cells [15]. Here, our study demonstrated that Smad7 functioned as an important anti-inflammatory regulator in human vascular endothelial cells.

We found that inhibition of the Smad7 expression mediated by miR-216a could enhance I*κ*B*α* phosphorylation, afterwards induce I*κ*B*α* degradation and activate NF-*κ*B response inflammation in the endothelial cells. This is consistent with previous studies that Smad7 blocks the recruitment of TGF-activated kinase and thereafter inhibits I*κ*B*α* phosphorylation and proteasomal degradation [13]. When endogenous miR-216a was suppressed by its synthetic inhibitor, the Smad7/I*κ*B*α* expression was rescued, which decreased endothelial inflammation and adhesive ability to monocytes. Moreover, in human carotid atherosclerosis plaque tissues, Smad7 mRNA expression level markedly decreased in the high miR-216a group in comparison with the low miR-216a group, suggesting a negative regulation of miR-216a on the Smad7 expression within human atherosclerotic lesions. In support with our study, Sandusky et al. reported that the Smad7 expression is significantly lower in the human coronary plaque endothelium than in normal vascular endothelium [16]. These data indicated that the reduction of the Smad7 expression mediated by miR-216a can activate vascular endothelial inflammatory responses and may accelerate atherosclerosis development.

As shown in our previous work, plasma miR-216a is specifically increased in aged patients with coronary artery disease and in patients with coronary artery vulnerable plaques [5, 8]. In this study, we provided new evidence that miR-216a was positively correlated with the IL1*β* expression in carotid atherosclerotic plaques, which could give more accurate information about miR-216a as a biomarker for vascular inflammation and atherosclerosis. Additionally, miR-216a is found to be upregulated both in the myocardium tissues and the plasma of heart failure patients compared with healthy controls [17], and the consistent result of plasma miR-216a in heart failure was recently reported by Ding et al. [18]. Thus, miR-216a may be a promising biomarker for vascular dysfunctions including endothelial senescence and inflammatory responses, atherosclerotic diseases, and heart failure.

## 5. Conclusions

In summary, we provided *in vitro* evidence that miR-216a promotes endothelial inflammation and enhances the adhesion ability of monocytes to endothelial cells by directly targeting the Smad7/I*κ*B*α* pathway. Consistently, the negative correlation between miR-216a and Smad7 is validated in human carotid plaques. These findings illuminate a new regulatory role of miR-216a in endothelial inflammation involving Smad7/I*κ*B*α* pathway, which suggests that miR-216a is a potential therapeutic target and a biomarker for atherosclerosis.

## Figures and Tables

**Table 1:** Clinical characteristics of patients who underwent carotid endarterectomy.

Characteristics	Low miR-216a group (*n* = 21)	High miR-216a group (*n* = 20)	*P*
Age, years	65.6 ± 8.8	62.9 ± 9.0	0.33
Male, *n* (%)	18 (85.7%)	17 (85.0%)	0.95
BMI, kg/m^2^	25.4 ± 3.2	24.7 ± 2.2	0.46
Hypertension, *n* (%)	18 (87.5%)	14 (70%)	0.22
Hyperlipidemia, *n* (%)	6 (31.6%)	10 (58.8%)	0.20
Diabetic mellitus, *n* (%)	14 (70.0%)	9 (45.0%)	0.11
Cigarette smoking, *n* (%)	13 (61.9%)	13 (68.4%)	0.21
Alcohol intake, *n* (%)	2 (9.5%)	7 (35.0%)	0.09
Systolic BP, mmHg	138 ± 19	139 ± 15	0.83
Diastolic BP, mmHg	82 ± 9	83 ± 10	0.76
Fasting glucose, mmol/L	6.50 ± 1.74	5.97 ± 1.44	0.31
Total cholesterol, mmol/L	4.91 ± 1.38	4.95 ± 1.16	0.93
Triglyceride, mmol/L	1.63 (1.31-2.06)	1.45 (1.26-1.91)	0.76
HDL-C, mmol/L	1.12 ± 0.52	1.05 ± 0.27	0.59
LDL-C, mmol/L	2.88 ± 1.21	2.89 ± 0.90	0.99
hsCRP, mg/L	8.92 ± 17.38	5.28 ± 5.92	0.50

BMI: body mass index; BP: blood pressure; HDL-C: high-density lipoprotein cholesterol; LDL-C: low-density lipoprotein cholesterol; hsCRP: high sensitive C-reaction protein.

## Data Availability

The data used to support the findings of this study are available from the corresponding author on reasonable request.
